# Effects of *HSD11B1* knockout and overexpression on local cortisol production and differentiation of mesenchymal stem cells

**DOI:** 10.3389/fbioe.2022.953034

**Published:** 2022-08-25

**Authors:** Angelique Kragl, Janosch Schoon, Ana Tzvetkova, Christoph Wenzel, Martina Blaschke, Wolfgang Böcker, Heide Siggelkow, Mladen V. Tzvetkov

**Affiliations:** ^1^ Institute of Pharmacology, Center of Drug Absorption and Transport (C_DAT), University Medicine Greifswald, Greifswald, Germany; ^2^ Center for Orthopaedics, Trauma Surgery and Rehabilitation Medicine, University Medicine Greifswald, Greifswald, Germany; ^3^ Institute of Bioinformatics, University Medicine Greifswald, Greifswald, Germany; ^4^ Human Molecular Genetics Group, Department of Functional Genomics, Interfaculty Institute of Genetics and Functional Genomics, University Medicine Greifswald, Greifswald, Germany; ^5^ Clinic of Gastroenterology, Gastrointestinal Oncology and Endocrinology, University Medical Center Göttingen, Göttingen, Germany; ^6^ MVZ Endokrinologikum Göttingen, Göttingen, Germany; ^7^ Department of Orthopaedics and Trauma Surgery, Musculoskeletal University Center Munich (MUM), University Hospital, Ludwig Maximilian University (LMU) Munich, Munich, Germany

**Keywords:** 11β-HSD1, osteoporosis, glucocorticoids, CRISPR-Cas9, targeted chromosomal integration

## Abstract

Exogenous glucocorticoids increase the risk for osteoporosis, but the role of endogenous glucocorticoids remains elusive. Here, we describe the generation and validation of a loss- and a gain-of-function model of the cortisol producing enzyme 11β-HSD1 (*HSD11B1*) to modulate the endogenous glucocorticoid conversion in SCP-1 cells — a model for human mesenchymal stem cells capable of adipogenic and osteogenic differentiation. CRISPR-Cas9 was successfully used to generate a cell line carrying a single base duplication and a 5 bp deletion in exon 5, leading to missense amino acid sequences after codon 146. These inactivating genomic alterations were validated by deep sequencing and by cloning with subsequent capillary sequencing. 11β-HSD1 protein levels were reduced by 70% in the knockout cells and cortisol production was not detectable. Targeted chromosomal integration was used to stably overexpress *HSD11B1*. Compared to wildtype cells, *HSD11B1* overexpression resulted in a 7.9-fold increase in *HSD11B1* mRNA expression, a 5-fold increase in 11β-HSD1 protein expression and 3.3-fold increase in extracellular cortisol levels under adipogenic differentiation. The generated cells were used to address the effects of 11β-HSD1 expression on adipogenic and osteogenic differentiation. Compared to the wildtype, *HSD11B1* overexpression led to a 3.7-fold increase in mRNA expression of lipoprotein lipase *(LPL)* and 2.5-fold increase in lipid production under adipogenic differentiation. Under osteogenic differentiation, *HSD11B1* knockout led to enhanced alkaline phosphatase (ALP) activity and mRNA expression, and *HSD11B1* overexpression resulted in a 4.6-fold and 11.7-fold increase in mRNA expression of Dickkopf-related protein 1 *(DKK1)* and *LPL*, respectively. Here we describe a *HSD11B1* loss- and gain-of-function model in SCP-1 cells at genetic, molecular and functional levels. We used these models to study the effects of endogenous cortisol production on mesenchymal stem cell differentiation and demonstrate an 11β-HSD1 dependent switch from osteogenic to adipogenic differentiation. These results might help to better understand the role of endogenous cortisol production in osteoporosis on a molecular and cellular level.

## Introduction

Osteoporosis is characterized by reduced bone mineral density leading to an increased risk of fractures, which results in an elevated morbidity and mortality (Abrahamsen et al., 2015; Ensrud et al., 2019). Every year, osteoporosis accounts for 8.9 million fractures worldwide. In Europe, 32 million people suffer from osteoporosis with women being more affected than men (Kanis et al., 2021).

Therapy with exogenous glucocorticoids is a major risk factor for osteoporosis (van Staa et al., 2002). In contrast, endogenous glucocorticoids play an essential role in bone homeostasis. Whereas systemic levels of cortisol are regulated centrally by the hypothalamic–pituitary–adrenal (HPA) axis, local cortisol levels are regulated by two isoenzymes: 11β-hydroxysteroid dehydrogenase 1 (11β-HSD1) and 2 (11β-HSD2) (Tannin et al., 1991; Agarwal et al., 1994; Albiston et al., 1994; Krozowski et al., 1995; Roland und Funder 1996; Bujalska et al., 1997; Cooper et al., 2000; Whorwood et al., 2002). 11β-HSD2 oxidizes the biologically active cortisol to inactive cortisone. Locally, cortisone can be re-activated to cortisol by 11β-HSD1 (gene name: *HSD11B1*) which is a crucial mechanism for mediation of the anti-inflammatory therapeutic effects of glucocorticoids (Fenton et al., 2021). 11β-HSD1 is most strongly expressed in liver (Tannin et al., 1991). Other relevant tissues include the adipose tissues, skeletal muscle and bone (Bujalska et al., 1997; Cooper et al., 2000; Whorwood et al., 2002). In bone, 11β-HSD1 is the predominant isozyme (Bland et al., 1999; Cooper et al., 2000) and plays an important role in osteoblast differentiation by providing the necessary glucocorticoid stimulus (Eijken et al., 2005). Notably, *HSD11B1* expression in osteoblasts and suppressed cortisol levels in patients evaluated for osteoporosis increase with age (Cooper et al., 2002; Siggelkow et al., 2014).

Mesenchymal stem cells (MSCs) are multipotent cells capable of differentiation into osteoblasts, adipocytes or chondrocytes. Osteoblasts are crucial for vital bone remodeling due to their ability to mineralize bone and to regulate osteoclast activity through paracrine signaling (Han et al., 2018). With age, apoptosis of human MSCs increases (Zhou et al., 2008) and differentiation into osteoblasts decreases in favor of differentiation into adipocytes (Moerman et al., 2004; Zhou et al., 2008).

SCP-1 cells are immortalized human MSCs that can be differentiated towards the osteogenic, adipogenic or chondrogenic lineage (Böcker et al., 2008). Previously we showed that transient *HSD11B1* overexpression and pharmacological inhibition of 11β-HSD1 affect osteogenic differentiation in SCP-1 cells by affecting cortisol production (Blaschke et al., 2021).

However, transient transfections and inhibition experiments limit the insight into the coherences of the overexpressed *HSD11B1* and changes in differentiation. Pharmacological inhibition may elicit off-target effects which potentially affect differentiation. With genetic inactivation or constant overexpression of *HSD11B1*, long-term effects of the absence or presence of 11β-HSD1 on differentiation can be monitored. Therefore, stable genetic modifications of MSCs resulting in complete inactivation or constant overexpression of *HSD11B1* would deliver a well-defined cell model leading to reliable and well interpretable results on differentiation capacity.

While the effects of exogenous glucocorticoids on bone homeostasis are well known [for a recent review see (Gado et al., 2022)], the role of endogenous cortisol and especially its local production by 11β-HSD1 need further investigations. Therefore, we here propose an *in vitro* approach to address the critical role of endogenous glucocorticoids on osteogenic and adipogenic differentiation of MSCs. We aimed 1) to use the CRISPR-Cas9 technology and targeted chromosomal integration in SCP-1 cells to generate a *HSD11B1* loss-of-function and gain-of-function model and 2) to use these models for analyzing the role of local cortisol levels on adipogenic and osteogenic differentiation of MSCs. With these models we aim to improve the understanding of the onset of age-related osteoporosis and thereby to identify new potential therapeutic strategies.

## Materials and methods

### Culturing and differentiation of SCP-1 cells

SCP-1 cells were cultured at 37°C in a humidified atmosphere (95%) with 5% CO_2_ in DMEM containing 4.5 g/l glucose, 0.58 g/I L-glutamine, 3.7 g/l NaHCO3 (without pyruvate) and supplemented with 10% fetal bovine serum, 100 U/ml penicillin and 100 μg/ml streptomycin. Medium and medium supplements were obtained from Thermo Fisher Scientific (Waltham, MA, United States).

For differentiation, 4 × 10^4^ SCP-1 cells per well were plated in 6-well plates. Adipogenic differentiation was induced by supplementing the culture media with 500 µM IBMX, 0.1 mg/ml insulin (Insuman^®^ Rapid), 200 µM indomethacin and 1 µM dexamethasone. For the first four days of adipogenic differentiation, only insulin was added. Osteogenic differentiation was induced by supplementing with 173 μM L-ascorbic acid 2-phosphate, 10 mM *β*-glycerol phosphate and 50 nM 1α,25-dihydroxy-vitamin D3. All stimulants were obtained from Sigma-Aldrich (St. Louis, MO, United States), except for insulin which was obtained from Sanofi (Paris, France). The differentiation medium was replaced every 3–4 days. For analyses of cortisol production over time and the influence of 11β-HSD1 on adipogenic and osteogenic differentiation, differentiation was performed in 24-well plates with 8 × 10^3^ SCP-1 cells per well. In the course of these experiments, dexamethasone was substituted by 5 µM cortisone (adipogenic differentiation) or was additionally added (osteogenic differentiation).

### Generation of *HSD11B1* knockout SCP-1 cells

The Alt-R™ CRISPR-Cas9 System (Integrated DNA technologies, Carolville, IA, United States) with the predesigned crRNAs Hs. Cas9. HSD11B1.1. AB (5′-CTA​CTA​CTA​TTC​TGC​AAA​CG-3′) targeting exon 2 and Hs. Cas9. HSD11B1.1. AA (5′-AGT​CAA​CTT​CCT​CAG​TTA​CG-3′) targeting exon 5 was chosen to knockout *HSD11B1*. SCP-1 cells were reverse transfected with RNP-complexes in 24-well plates as described before (Schwefel et al., 2018) with the following modifications: the medium was changed to normal culture medium after 24 h. As the cells reached confluence, they were detached and one half of the cell suspension was transferred into a 6-well plate to 2 ml culture medium. The other half was used for DNA isolation. Upon reaching confluence in the 6-well plate, the cells were transferred into T25 flasks. Subsequently, the cells were diluted and transferred into 96-well plates applying the low-density dilution method. As our preliminary analyses showed poor clonal growth, the cells were diluted in the following densities: 0.8 cells/well, 2.5 cells/well and 5.0 cells/well. Both single cell clones and clones arisen from 2–3 cells were further expanded in 12-well plates and T25 flasks and further analyzed.

For DNA isolation, the pelleted cells were resuspended in 50 µl QuickExtract™ DNA Extraction Solution (Epicentre, Middleton, WI, United States) and were heated at 65°C for 10 min, followed by heating at 98°C for 5 min. The isolate was diluted 1:5 with sterile water.

Potential off-target sites were chosen based on their ranking in the IDT and CCTop off-target prediction. For CCTop analyses (CCTop, RRID:SCR_016890), the default settings were used (PAM type: NGG, target site length: 20 bp, core length: 12 bp, max. Total mismatches: 4, max. Core mismatches: 2). Sequences of predicted off-target sites were determined by capillary Sanger sequencing.

### Generation of *HSD11B1* overexpressing SCP-1 cells

*HSD11B1* was stably overexpressed in SCP-1 cells applying the Flp-In™ System (Invitrogen, Carlsbad, CA, United States). The coding sequence of *HSD11B1* was amplified from the plasmid pcDNA3.1-HSD11B1 and was subsequently cloned into the pcDNA5/FRT Expression Vector (Invitrogen) using EcoRV and HindIII restriction sites. The Rapid DNA Ligation Kit (Thermo Fisher Scientific) was used for ligation.

To generate the Flp-In™ host cell line, 2 × 10^5^ SCP-1 cells were plated and cultured for 24 h. The cells were transfected with 4 µg of the linearized pFRT/*lac*Zeo plasmid using 12 µl FuGENE 6 (Promega, Madison, WI, United States), in a 6-well plate. After 24 h, the culture medium was changed to complete medium. Upon reaching 100% confluence, the cells were transferred into cell culture dishes (100 mm) and after 24 h the medium was replaced by selection medium (complete medium supplemented with 400 μg/ml Zeocin™). When resistant clones became visible, they were transferred into 12-well plates. The medium was changed every 3–4 days. When the cells reached confluence, they were sequentially transferred into a 6-well plate, a T25 flask and finally into a T75 flask. Cells were cultured in selection medium until cryopreservation. Success of transfection was determined by *β*-galactosidase activity with the *β*-Gal Assay Kit (Invitrogen, Carlsbad, CA, United States).

Stable transfection of SCP-1/FRT cells with the *HSD11B1* encoding Expression Vector (pcDNA5/FRT:*HSD11B1*) was performed in a 6-well plate as described above with the following modifications: the cells were transfected with 400 ng pcDNA5/FRT:*HSD11B1* and 3.6 µg pOG44 using 12 µl FuGENE 6 (Promega). One well was transfected with 400 ng pcDNA5/FRT:*HSD11B1* and 3.6 µg GFP-tpz and served as a transfection control. As selection antibiotic hygromycin was used with a final concentration of 200 μg/ml. After transfer into 12-well plates, a reduced hygromycin concentration of 100 μg/ml was applied. A reduced hygromycin concentration of 50 μg/ml was used in the 6-well plate, T25 flask and T75 flask.

### Validation PCRs

PCRs for the genetic validation of overexpressing cells were performed as described before (Saadatmand et al., 2012). Additionally, a third PCR was performed to check for multiple integration (Supplementary Table S1). Amplification was performed using the QIAGEN Multiplex PCR Kit (Qiagen, Hilden, Germany) and the following reaction conditions: 95°C for 15 min, followed by 35 cycles of 94°C for 30 s, 58°C for 1 min 30 s, 72°C for 2 min, and finally 72°C for 10 min.

### T7 endonuclease I assay

Efficiency of CRISPR-Cas9 mediated gene editing was determined by T7EI (New England Biolabs, Ipswich, MA, United States) digest of annealed PCR amplicons of the regions of interest (Supplementary Table S1). For *CCM3*, the primers for the T7EI assay were kindly provided by S. Spiegler and U. Felbor (Department of Human Genetics, University Medicine Greifswald) (Schwefel et al., 2018). Following gel electrophoresis, the fragment pattern was analyzed and the DNA amount of each fragment was determined by measuring the integrated intensity applying Fiji (Schindelin et al., 2012). The efficiency of CRISPR-Cas9 treatment was validated by estimating the indel occurrence as described before (Ran et al., 2013).

### TOPO^®^ TA cloning

Cloning was performed with the amplification products of the T7EI PCR and TOPO TA Cloning^®^ Kit (Invitrogen, Carlsbad, CA, United States) according to the manufacturer’s protocol. The generated TOPO clones were sequenced using the BigDye™ Terminator v1.1 Cycle Sequencing Kit (Thermo Fisher Scientific).

### Deep sequencing

For deep sequencing, a Next Generation Sequencing (NGS) approach was used. The region of interest was amplified by PCR (Supplementary Table S1). Purification and sequencing was performed as described before (Römer et al., 2021) with the primers listed in Supplementary Table S1 and the MiSeq Reagent Nano Kit v2 (500-cycles) (Illumina, Inc. San Diego, United States) on the MiSeq System (Illumina) with paired-end reads.

To perform the following three steps we used the software tool VSEARCH version 2.14.2 (Rognes et al., 2016). First, we merged the paired-end reads using default parameters. We then kept only reads with maximum number of expected errors 1 and length of at least 200 bp. Finally, the reads with an identity of 99% in each clone were clustered together. The centroid sequence from each cluster that contained more than 50 reads was aligned against the reference sequence (NG_012081.1) to analyze sequence modifications.

### Gene expression analysis

For validation experiments, RNA was isolated with the RNeasy *Plus* Mini Kit (Qiagen). Approximately 1 × 10^6^ cells were pelleted and 350 µl RLT *Plus* Buffer supplemented with 1% *β*-mercaptoethanol were added. The suspension was either stored at −20°C or directly submitted to the QIAcube robot.

RNA of differentiated SCP-1 cells was isolated with a modified protocol of the single-step method (Chomczynski und Sacchi 1987) using TRIzol™ Reagent (Thermo Fisher Scientific) and chloroform. Per well of a 24-well plate 500 µl TRIzol™ were added and the lysates of two wells were pooled and stored at −20°C. Lysates were thawed on ice and 200 µl chloroform were added and mixed with the lysates by shaking. After a 10-min incubation on ice, the samples were centrifuged at 12,000 *g* and 4°C for 10 min. The aqueous phase was carefully transferred into a fresh 1.5 ml reaction tube. The RNA was precipitated by addition of 500 µl ice-cold isopropanol followed by incubation at −20°C for 10–30 min, depending on the expected yield. By centrifugation at 12,000 *g* for 20 min, the RNA was pelleted. The supernatant was removed and the RNA was washed with 500–800 µl ice-cold 70% ethanol, depending on the pellet size. The centrifugation was repeated and the ethanol was completely removed with a pipette. The pellet was allowed to dry at room temperature until it became transparent. To dissolve the RNA, 25–30 µl sterile water were added followed by incubation at 60°C for 5 min.

Isolated mRNA was reverse transcribed into cDNA applying the High Capacity cDNA Reverse Transcription Kit (Thermo Fisher Scientific). Gene expression was analyzed by RT-qPCR using predesigned TaqMan^®^ Gene Expression Assays (Thermo Fisher Scientific): Hs01060665_g1 (*ACTB*), Hs01029144_m1 (*ALPL*), Hs00183740_m1 (*DKK1*), Hs00194153_m1 (*HSD11B1*), Hs00173425_m1 (*LPL*), Hs99999910_m1 (*TBP*). For graphical representation, the Ct values were converted into relative expression by applying the ΔCt method. The expression was calculated as gene of interest transcripts per 1,000 housekeeping gene transcripts. Gene expression of *HSD11B1* in differentiated SCP-1 cells was normalized to *ACTB* expression, as *ACTB* is the most stably expressed housekeeping gene in differentiated SCP-1 cells.

### Protein quantification by Western Blot

SCP-1 cells were either differentiated adipogenically for 14 days or cultured without stimulants. The cells were detached and pelleted by centrifuging at 300 *g* for 3 min. The cell pellets were washed with PBS and subsequently resuspended in 30 µl 5 mM Tris-HCl (pH 7.4) supplemented with 100 μM PMSF, 1 µM leupeptin and 3 μg/ml aprotinin. After five freeze-thaw cycles, the lysate was transferred into a 1.5 ml tube and centrifuged at 100,000 *g* and 4°C for 30 min. The supernatant was discarded and the pelleted membranes were resuspended in 20 µl 5 mM Tris-HCl supplemented with protease inhibitors (see above). The pelleted membranes were further disrupted by passing through a 27 G needle. The protein amount was quantified using a BCA assay (Thermo Fisher Scientific) and 50 µg protein were mixed with 4x Laemmli Buffer (20% glycerol, 100 mM Tris-HCl pH 6.8, 0.02% Orange G, 6% SDS, 2% DTT) supplemented with 2-mercaptoethanol (1:10). The proteins were gently denatured at 37°C for 30 min. SDS-PAGE was performed with a 12.5% separation gel and a 4% stacking gel. The proteins were transferred to a nitrocellulose membrane (GE Healthcare, Chicago, IL, United States) applying the tank blot procedure on ice using pre-cooled Towbin buffer (Towbin et al., 1979) with increased methanol content (30%) at a constant current of 370 mA. The membranes were blocked in TBST (25 mM Tris, 136 mM NaCl, 3 mM KCl, 0.04% Tween 20) with 10% (v/v) FCS and as both primary antibodies used originate from rabbit, the membrane was cut horizontally to circumvent cross-detection with the secondary antibody. Incubation with anti-HSD11B1 (1:1,000; Abcam Cat# ab157223, RRID:AB_2630342) and anti-Na^+^/K^+^-ATPase (1:2000; Abcam Cat# ab76020, RRID:AB_1310695) followed at 4°C in a tube rotator for 48 h and overnight, respectively. The secondary antibody was incubated at room temperature on a shaking platform for 1 h (1:20,000; LI-COR Biosciences Cat# 925-68071, RRID:AB_2721181; LI-COR Biosciences Cat# 925-32211, RRID:AB_2651127). All antibodies were diluted in TBST supplemented with 0.05% sodium azide. Anti-HSD11B1 binds at the C-terminus of the protein. The blot was developed with the Odyssey^®^ CLx (LI-COR) by detection of fluorescence at 700 nm (HSD11B1) or 800 nm (Na^+^/K^+^-ATPase). Signals were quantified with the Image Studio™ software (LI-COR).

### Protein quantification by targeted proteomics

Targeted proteomics were performed as described before (Meyer et al., 2020). Here, wildtype, *HSD11B1* knockout and overexpressing SCP-1 cells were differentiated adipogenically for 14 days. For the LC-MS/MS measurement, an injection volume of 20 µl was used. Solvent A (0.1% formic acid in acetonitrile) and Solvent B (0.1% formic acid in water) were mixed applying a gradient over time (Supplementary Table S2). The peptide QEEVYYDSSLWTTLLIR (JPT Peptide Technologies GmbH, Berlin, Germany) was chosen for detection of 11β-HSD1, targeting the C-terminus at 253–269 aa (UniProtKB P28845). For normalization, Na^+^/K^+^-ATPase protein level was detected with the peptide LSLDELHR (Thermo Fisher Scientific). The MS detection parameters applied are listed in Supplementary Table S3. The measurement was performed in three replicates. The peak areas were automatically determined by the Analyst 1.6.3 software (Sciex, Darmstadt, Germany). Peak areas of 11β-HSD1 were determined for wildtype, knockout and overexpressing cells and normalized to the respective peak area of Na^+^/K^+^-ATPase, which allows a relative quantitation of 11β-HSD1. For 11β-HSD1 four and for Na^+^/K^+^-ATPase three mass transitions were analyzed. The relative 11β-HSD1 protein level was calculated as ratio of the mean of the four 11β-HSD1 measurements and the mean of the three Na^+^/K^+^-ATPase measurements.

### Measurement of 11β-HSD1 activity

To functionally validate the knockout and overexpressing SCP-1 cell lines, wildtype, *HSD11B1* knockout and overexpressing cells were adipogenically differentiated for 14 days. On day five and twelve of differentiation, cells were stimulated with 5 µM cortisone. To this end, FCS was substituted by BSA which was diluted in the medium to a final concentration of 0.1%. As an additional control, cell-free medium was incubated. After 48 h, the medium was removed and analyzed.

To quantify extracellular cortisol levels, the medium was prepared by heavily mixing 200 µl with 800 µl 100% acetonitrile supplemented with 50 ng/ml cortisol-d4 and centrifuged at 16,000 *g* for 15 min to pellet the debris. Subsequently, 350 µl of the supernatant were evaporated to dryness under nitrogen flow at 40°C. The dried pellet was resuspended in 200 µl 0.1% formic acid (50% 0.2% formic acid, 50% acetonitrile + methanol (6 + 1)) and centrifuged at 16,000 *g* for 5 min. Fifteen µl were injected into the LC-MS/MS system which consisted of an API 4000 QTRAP^®^ tandem mass spectrometer (AB Sciex Ontario, Canada) with ESI interface coupled to a Shimadzu Nexera X2 UHPLC system with LC 30AD pumps and SIL 30AC autosampler (Shimadzu, Kyoto, Japan) (Supplementary Table S4). Samples were separated using a Brownlee SPP RP-Amide (4.6 × 100 nm, 2.7 µm particle size) column (Perkin Elmer, Waltham, MA, United States) and a flow rate of 500 μl/min. Solvent A (0.1% formic acid in 90% acetonitrile + methanol (6 + 1)) was mixed in equal volumes with solvent B (0.1% formic acid in water).

### Staining and quantification of lipid droplets

Cells were fixed with 4% paraformaldehyde. Fixed cells were stored in PBS at 4°C. Lipid droplets were stained with Nile Red, nuclei were stained with DAPI as described before (Rakow et al., 2016; Andrzejewska et al., 2019). The cells were incubated with 400 µl staining solution (1 μg/ml Nile Red and 1 μg/ml DAPI in PBS; both from Sigma-Aldrich, St. Louis, MO, United States) in the dark at room temperature for 15 min. Thereafter, the cells were washed and overlaid with PBS. Fluorescence and background for Nile Red and DAPI were detected at 538 nm after excitation at 485 nm and at 454 nm after excitation at 364 nm, respectively, in a Tecan infinite M200 microplate reader (Tecan Group Ltd. Männedorf, Switzerland). For both dyes, the background values were subtracted from the fluorescence values and Nile Red measurements were normalized on DAPI. Microphotographs were taken using a LSM780 with a 10x EC PlnN 10X/0.3 DICI M27 objective.

### Alkaline phosphatase assay

The ALP assay was performed as described before (Ode et al., 2013). After washing with 400 µl PBS and 500 µl AP Buffer (100 mM NaCl, 100 mM Tris, 1 mM MgCl_2_, pH 9.0), 250 µl AP Buffer and 250 µl *p*-nitrophenyl phosphate (*p*NPP) solution (1 mg/ml in 1 M diethanolamine, pH 9.8; Sigma-Aldrich) were added. After a 10-min incubation at 37°C in a CO_2_-incubator, the reaction was stopped by addition of 500 µl 1 N NaOH. The absorbance at 405 nm was determined in a microplate reader and the concentration of *p*-nitrophenol (*p*NP) (i.e., consumed *p*NPP) was determined using the Beer-Lambert equation.

### Statistical methods

Descriptive statistics and data plotting were performed with GraphPad Prism v.5.01. One-way ANOVA with post-hoc Tukey’s Test for multiple comparisons was performed with IBM SPSS Statistics v.26. Results were called significant when *p* ≤ 0.05.

## Results

### Generation and genetic validation of *HSD11B1* knockout cells

**FIGURE 1:**
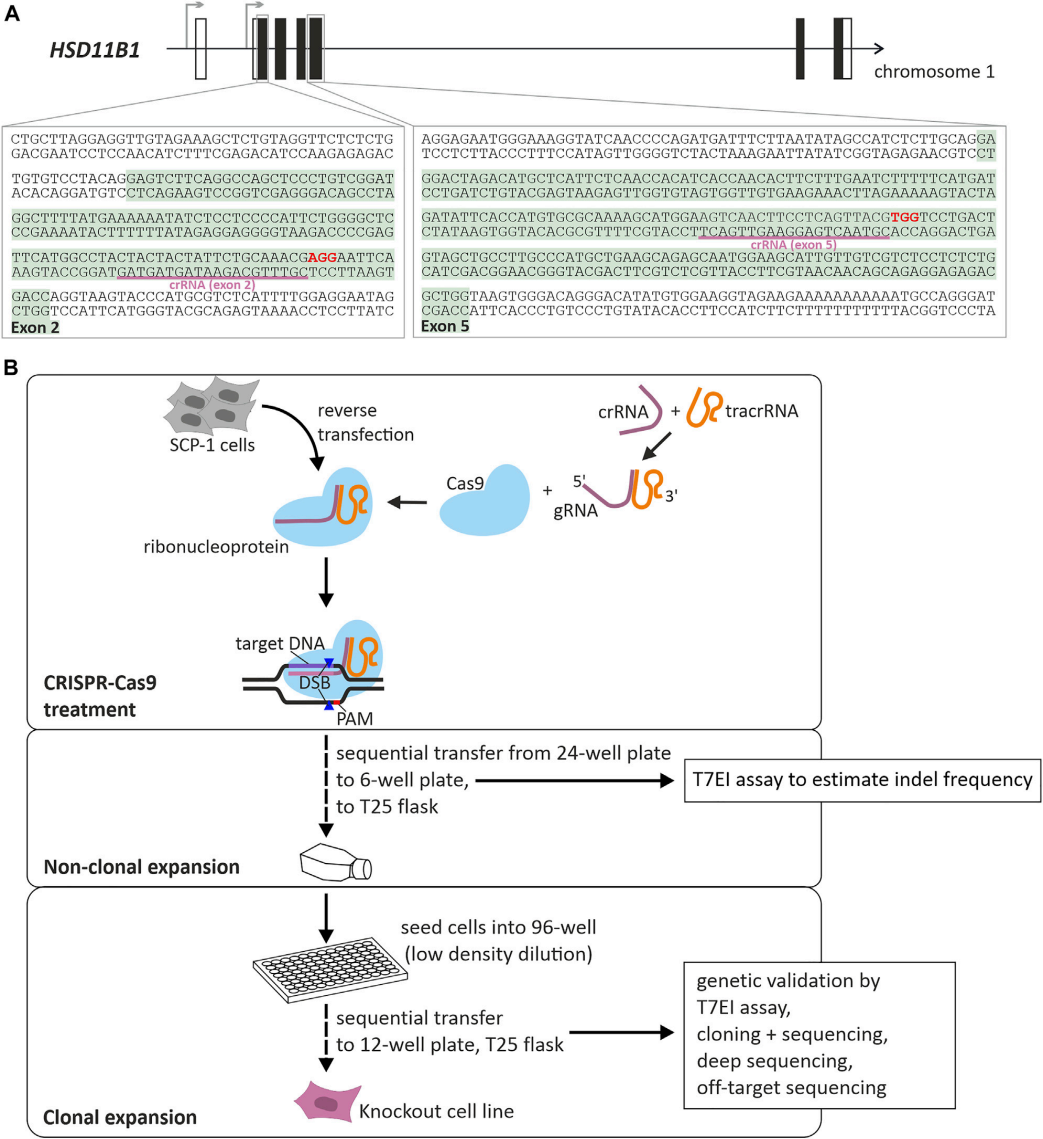
Generation of *HSD11B1* knockout SCP-1 cells using CRISPR-Cas9 mediated gene editing. **(A)** Schematic representation of the localization of the targeted regions within *HSD11B1*. Two crRNAs were applied, one targeting exon 2, the other targeting exon 5. The binding sites of the crRNAs is underlined, the PAM sites are shown in bold print. **(B)** Workflow and components of CRISPR-Cas9 mediated gene editing applying the ribonucleoprotein approach of IDT. A crRNA is designed so as to contain a sequence complementary to the target site. It is mixed with a tracrRNA to form the gRNA. This gRNA forms a ribonucleoprotein with the Cas9 endonuclease. After transfection of target cells, Cas9 will be directed to the target site and will create a double strand break (DSB) upstream of the PAM site.

**FIGURE 2:**
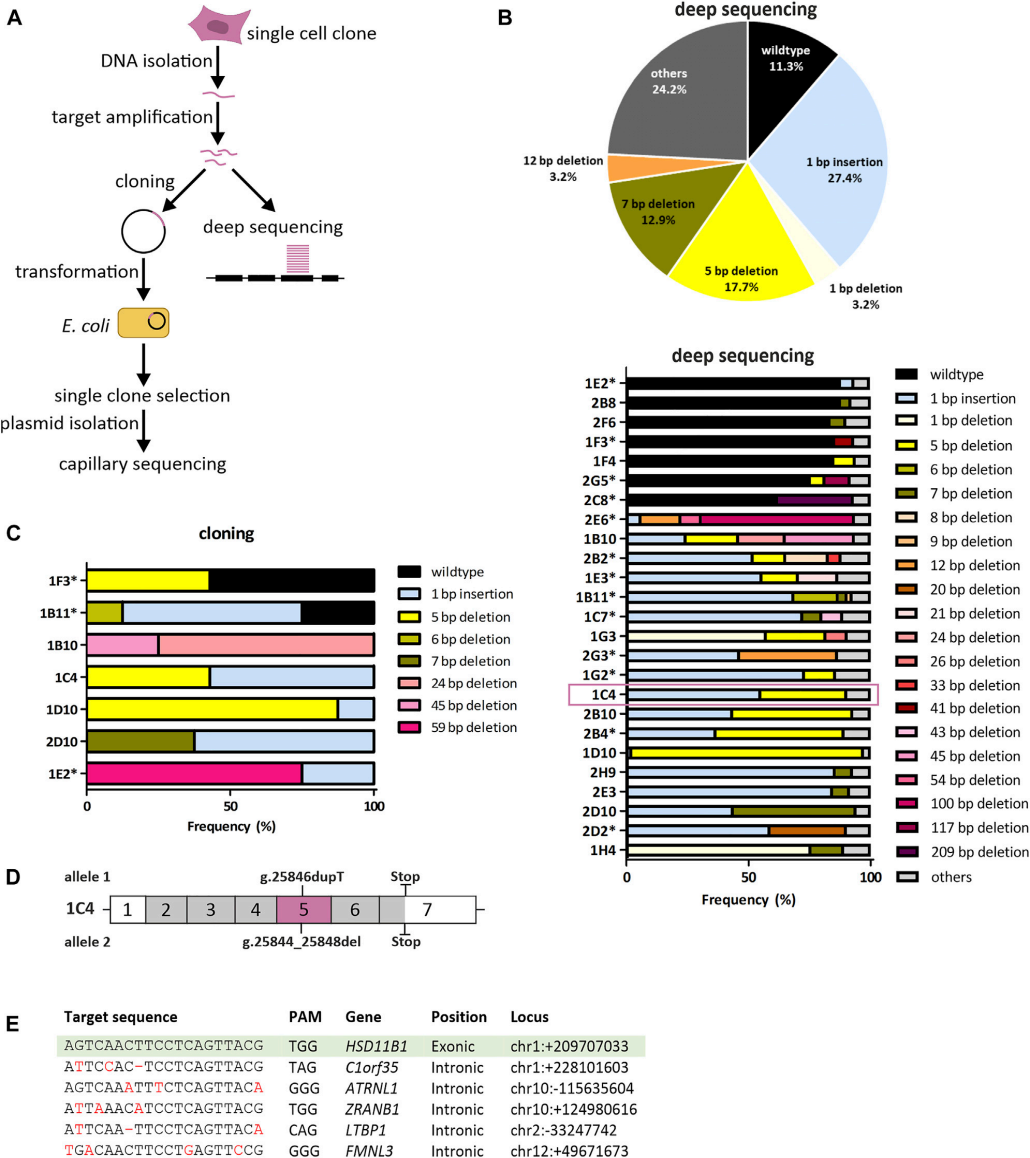
Genetic validation of *HSD11B1* knockout SCP-1 cells generated using the crRNA targeting exon 5. **(A)** Sequencing strategy to identify sequence modifications in the CRISPR-Cas9 treated cells. **(B)** Summarized allele frequencies of sequence modifications detected by NGS. Others represents additional modifications that had a frequency of less than 2% (top). Read frequencies of sequence modifications across all clones determined by NGS. Others represents the clusters that were not analyzed in detail due to a read number <50 (bottom). Asterisks indicate clones with potential origin from more than one cell. **(C)** Read frequencies of sequence modifications determined by cloning and subsequent capillary sequencing. **(D)** Schematic representation of sequence modifications and consequences in clone 1C4. Positions given are based on the reference sequence NG_012081.1. **(E)** Potential off-target sites of the applied *HSD11B1* exon 5 crRNA predicted by IDT and CCTop. Mismatches in the target sequence are marked red, hyphens indicate gaps. Sequences of the respective sites were controlled by capillary sequencing.

We applied CRISPR-Cas9 mediated gene editing to knockout *HSD11B1* in SCP-1 cells. Two independent targets were used, one in exon 2 and one in exon 5 (Figure 1A). The efficiency of CRISPR-Cas9 mediated gene editing was controlled using T7EI assays and the cells were diluted to propagate single cell clones (Figure 1B; Supplementary Figure S1). Following the clonal selection, a T7EI assay was applied to select 38 clones for further analyses: 13 clones where exon 2 was targeted and 25 clones where exon 5 was targeted. The knockout in the chosen clones was validated by cloning with subsequent capillary sequencing and by deep sequencing using a Next Generation Sequencing technology (Figure 2).

Deep sequencing was performed for all clones with an average depth of 2,431 reads (range 555–8,016). Following clustering to account for sequencing errors, an average of 2.3 clusters per clone were analyzed (range 1–6).

The knockout efficiency was substantially better when exon 5 than when exon 2 was targeted. Among the exon 5 clones analyzed, 88.7% did not contain the wildtype sequence. The most predominant modifications detected with deep sequencing in exon 5 were a 1 bp insertion, 5 bp deletion and 7 bp deletion (Figure 2B). In parallel, we analyzed seven exon 5 clones in a “classical” low-throughput analyses by cloning the targeted region in bacteria and resequencing single bacterial clones (mean number of bacterial clones sequenced: 7.3 per cell clone, range 4–8). In five out of seven clones analyzed no wildtype sequences were detected (Figure 2C). Three clones showed similar distribution of the genetic changes both in the capillary and in the deep sequencing: 1C4, 1D10 and 2D10. The clone 1C4 was chosen for further analyses.

The validation of exon 2 clones revealed that eight out of thirteen clones analyzed (62%) contained either completely or to more than 40% wildtype reads. Further three clones showed changes that did not lead to a frameshift (Supplementary Figure S2). Only two clones (1B7, 1F4) indicated a frameshift. However, single wildtype reads were found also in these clones that might would have become predominant after several passages. Hence, no exon 2 clones were further used.

We chose the clone 1C4 for subsequent experiments. This clone had a 1 bp insertion originating from a duplication of T at position 25846 (NG_012081.1) and a 5 bp deletion occurring at positions 25844–25848 (NG_012081.1). Both nucleotide changes caused frameshifts leading to missense amino acid sequences after codons 146 and 145 (Supplementary Figure S3), which resulted in premature stop codons at amino acids 257 and 259, respectively (Figure 2D).

To exclude off-target effects, we sequenced five gene loci in the human genome that were predicted due to sequence homology to be the most probable off-targets of the used crRNA (Figure 2E). None of them showed alterations compared to the wildtype sequence in the clone 1C4 suggesting no off-target artefacts of the CRISPR-Cas9 mediated gene editing.

### Generation and genetic validation of *HSD11B1* overexpressing cells

**FIGURE 3:**
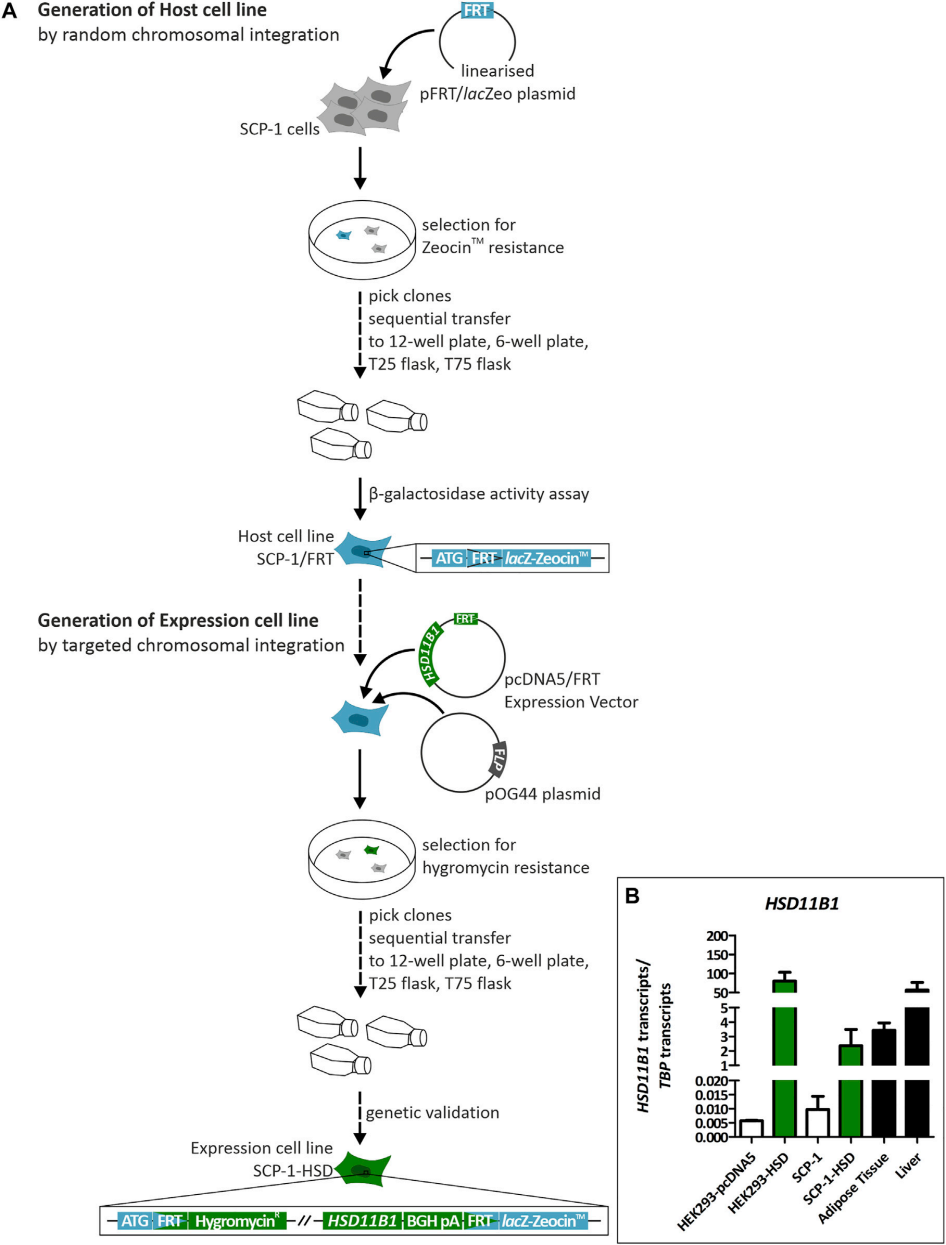
Generation and genetic validation of stably *HSD11B1* overexpressing SCP-1 cells using the Flp-In™ system. **(A)** The host cell line is created by transfection with the pFRT/*lac*Zeo plasmid. The FRT site is randomly integrated. The expression cell line is generated by transfection with the pcDNA5/FRT expression vector carrying *HSD11B1* and the pOG44 plasmid expressing *Flp* recombinase. **(B)** *HSD11B1* mRNA expression in *HSD11B1* overexpressing HEK293 and SCP-1 cells compared to expression in human adipose tissue and liver. As controls untransfected SCP-1 cells and HEK293 cells transfected with the empty pcDNA5 vector were used. Shown are the HEK293-HSD clone I/6 and the SCP-1 clone I/1. Shown are means ± SEM of 2–5 biological replicates.

We used targeted chromosomal integration to generate SCP-1 cells stably overexpressing *HSD11B1*. To this end, first, the target FRT site was integrated into SCP-1 cells to create the host cell line (Figure 3). The resulting SCP-1/FRT cells were used to integrate the complete ORF of the human *HSD11B1*. Flp-In™ T-REx 293 (HEK293) were also transfected with *HSD11B1* as a control. Correct chromosomal integration was validated by PCR (Supplementary Figure S4) and the *HSD11B1* expression levels were determined by RT-qPCR (Figure 3B). Compared to the untransfected cells, *HSD11B1* expression increased 243-fold in the stably transfected SCP-1 cells and 13,974-fold in the HEK293 cells (Figure 3B). The resulting *HSD11B1* expression in the stably transfected SCP-1 cells (2.4 transcripts per *TBP* transcript) were comparable with the *HSD11B1* expression levels in human adipose tissue. *HSD11B1* expression in the overexpressing HEK293 clone (80.3 transcripts per *TBP* transcript) was comparable with the levels in the liver (Figure 3B).

### Functional validation of *HSD11B1* knockout and overexpression in SCP-1 cells

**FIGURE 4:**
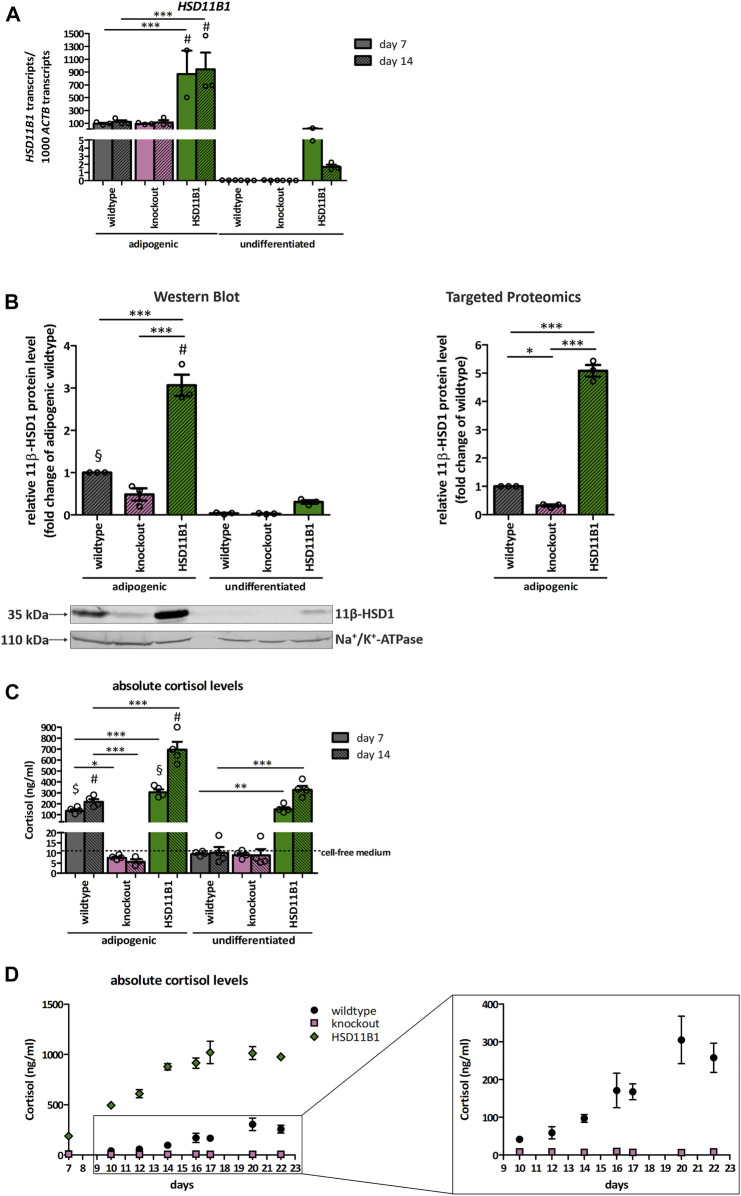
Functional validation of the *HSD11B1* knockout and overexpression in SCP-1 cells after adipogenic differentiation. Functional validation has been performed by analyzing **(A)** mRNA expression, **(B)** protein expression, 11β-HSD1 activity measuring **(C)** extracellular cortisol levels and **(D)** extracellular cortisol levels over time. **(A)** *HSD11B1* mRNA expression was determined by RT-qPCR. Shown are means ± SEM of 2–3 biological replicates. **(B)** Protein expression after 14 days of adipogenic differentiation as determined by Western Blot analysis (left) and Targeted Proteomics (right). A representative Western Blot is given. In both approaches, 11β-HSD1 protein levels were normalized to Na^+^/K^+^-ATPase. Cells were differentiated for 14 days. Shown are means ± SEM of 3 Western Blot quantifications from two independent differentiation experiments and 3 Targeted Proteomics measurements from a single differentiation experiment. **(C)** 11β-HSD1 activity was determined by cortisol measurements after a 48-h stimulation with cortisone. Shown are means ± SEM of 3–4 biological replicates. **(D)** 11β-HSD1 activity was determined by cortisol measurements in samples taken with every medium replacement. Absolute cortisol levels in days are depicted. Shown are means ± SEM of 3–4 biological replicates. Cells were differentiated with IBMX, indomethacin, insulin, and dexamethasone **(A–C)** or cortisone **(D)**. *p ≤ 0.05, **p ≤ 0.01, ***p ≤ 0.001; $ *p* ≤ 0.05, §*p* ≤ 0.01, #*p* ≤ 0.001 compared to respective undifferentiated control, determined by one-way ANOVA with post-hoc Tukey’s Test.

To validate the *HSD11B1* knockout and overexpression at functional level, we analyzed mRNA and protein expression, and cortisol production during adipogenic differentiation of SCP-1 cells. Adipogenic differentiation was chosen as this resulted in the highest increase in endogenous *HSD11B1* expression in SCP-1 cells. *HSD11B1* expression increased 3,000-fold in wildtype SCP-1 cells already on day 7 and remained stably on the same level on day 14 of differentiation (Figure 4A). Compared to the wildtype cells, *HSD11B1* expression in overexpressing cells was more than 100-fold higher in the undifferentiated, 9.4-fold higher at day 7 (*p* < 0.001) and 7.9-fold higher at day 14 of differentiation (*p* < 0.001). *HSD11B1* expression was not diminished in the *HSD11B1* knockout cells.

11β-HSD1 protein expression was analyzed by Western blot and targeted proteomics at day 14 of adipogenic differentiation (Figure 4B). With both approaches, the 11β-HSD1 protein level in overexpressing cells significantly increased (3-fold in Western blot and 5-fold in targeted proteomics) compared to wildtype cells (*p* < 0.001 and *p* < 0.001). In knockout cells, 11β-HSD1 protein levels were reduced by 50% (Western Blot) and by 70% (targeted proteomics, *p* < 0.05) when compared to wildtype cells. In undifferentiated cells, 11β-HSD1 protein was only detectable in overexpressing cells.

More importantly, the functional effects of *HSD11B1* overexpression and knockout were clearly observed when we analyzed cortisol production in medium supplemented with cortisone. The concentration of extracellular cortisol of wildtype cells increased 14-fold, upon seven days of differentiation (Figure 4C). The extracellular cortisol level increased constantly from day 10 and reached its maximum of 305 ng/ml on day 20 (Figure 4D). In *HSD11B1* overexpressing cells, the cortisol production exceeded the production in the wildtype cells by at least 3.3-fold at any time point of the measurements. A constant increase of the cortisol level was detected, with the highest cortisol concentration of 1,020 ng/ml reached at day 17 of the differentiation. Importantly, despite lack of changes in *HSD11B1* mRNA levels and the detection of trace amounts of 11β-HSD1 protein, no cortisol production was detectable at any time point of differentiation of the *HSD11B1* knockout cells (Figures 4C,D).

### Analyses on the effects of endogenous cortisol on adipogenic and osteogenic differentiation

**FIGURE 5:**
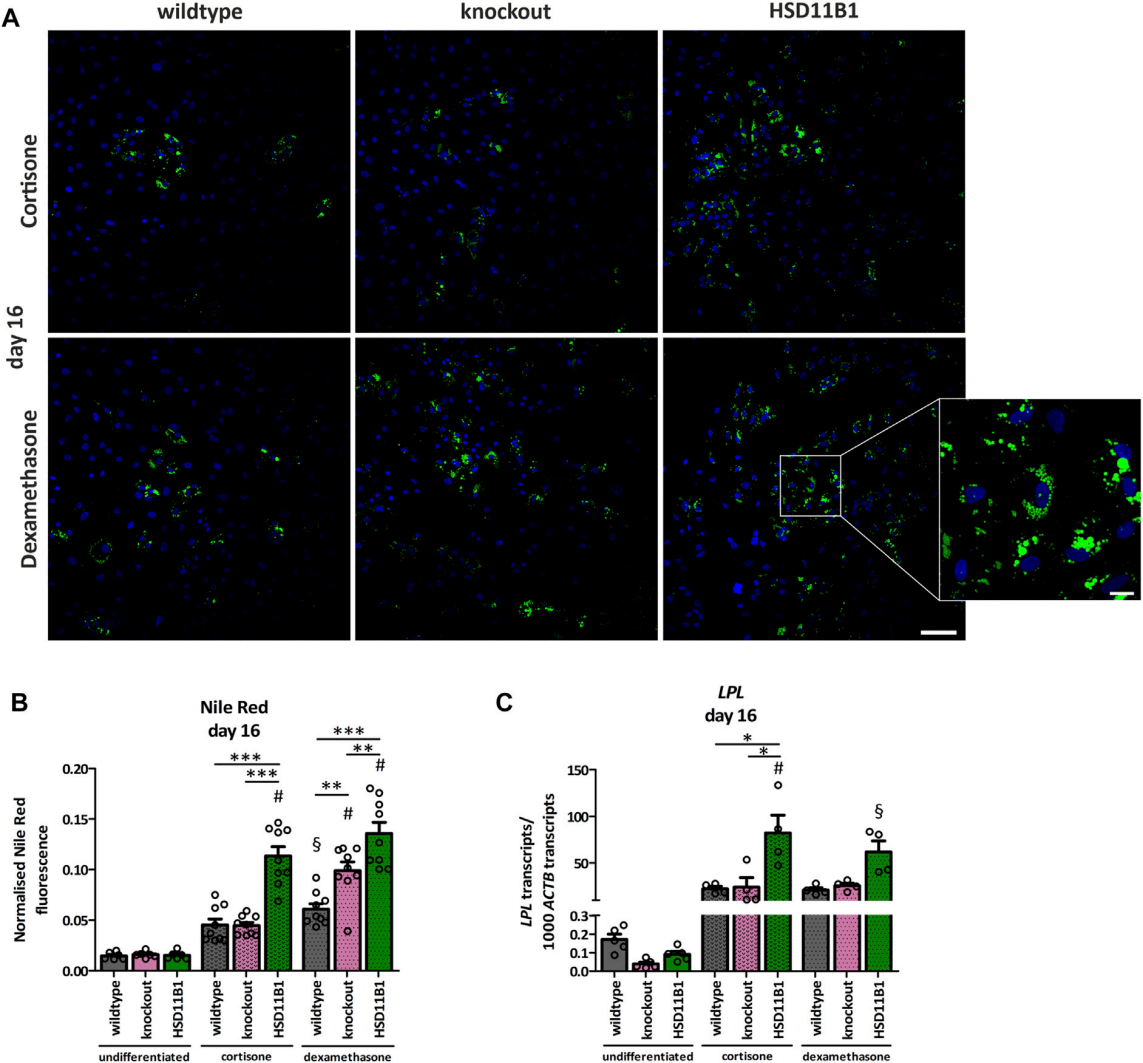
Analyses of the effects of *HSD11B1* knockout and overexpression on adipogenic differentiation. **(A)** Lipid droplets were visualized by Nile Red staining, nuclei were stained with DAPI. Representative images for each cell line are given. Scale bar: 100 µm. Zoom into overexpressing cells differentiated with dexamethasone (zoom factor: 5.1x, scale bar: 20 µm). **(B)** Nile Red was quantified directly after staining by fluorescence measurement and normalized on DAPI fluorescence. Shown are means ± SEM of 6 wells of undifferentiated and 9 wells of differentiated cells from 3 independent differentiation experiments. **(C)** mRNA expression of the adipogenic marker *LPL*. Shown are means ± SEM of 5 (undifferentiated) and 4 (differentiated) independent differentiation experiments. Cells were differentiated with IBMX, indomethacin, insulin, and cortisone or dexamethasone. *p ≤ 0.05, **p ≤ 0.01; §*p* ≤ 0.01, #*p* ≤ 0.001 compared to the respective undifferentiated cells, determined by one-way ANOVA with post-hoc Tukey’s Test.

The here generated cell models were used to study the influence of endogenously produced cortisol on adipogenic and osteogenic differentiation. To evaluate the effects of *HSD11B1* mediated cortisol production on adipogenic differentiation, lipid droplets were visualized and quantified using Nile Red staining. At day 16 of adipogenic differentiation, a significant increase in lipid droplets was detected in all three cell lines: wildtype, *HSD11B1* knockout and overexpressing (Figures 5A,B), demonstrating a successful differentiation of SCP-1 cells towards the adipogenic lineage. The overexpressing cells showed the highest lipid accumulation. After 16 days of adipogenic differentiation, in a protocol where dexamethasone was substituted with cortisone, Nile Red fluorescence increased 3.1-fold in the wildtype and 7.5-fold in the overexpressing cells (*p* < 0.001, Figure 5B).

Additionally, the success and the extent of adipogenic differentiation in the three cell lines were analyzed by gene expression analyses of the adipogenic marker gene Lipoprotein Lipase (*LPL*). The increase in *LPL* expression on day 16 of differentiation was significantly stronger in the overexpressing cells (915-fold) compared to the increase in the wildtype cells (132-fold, *p* < 0.05; Figure 5C).

The *HSD11B1* knockout did not lead to a decrease in adipogenic differentiation, neither in terms of *LPL* expression nor in accumulation of lipid droplets (Figures 5B,C). The higher adipogenic differentiation efficiency was observed in the *HSD11B1* overexpressing cells also under a control stimulation with dexamethasone (Figures 5A–C). These experiments suggest that *HSD11B1* overexpression increases adipogenic differentiation of SCP-1 cells independent of cortisone stimulation.

**FIGURE 6:**
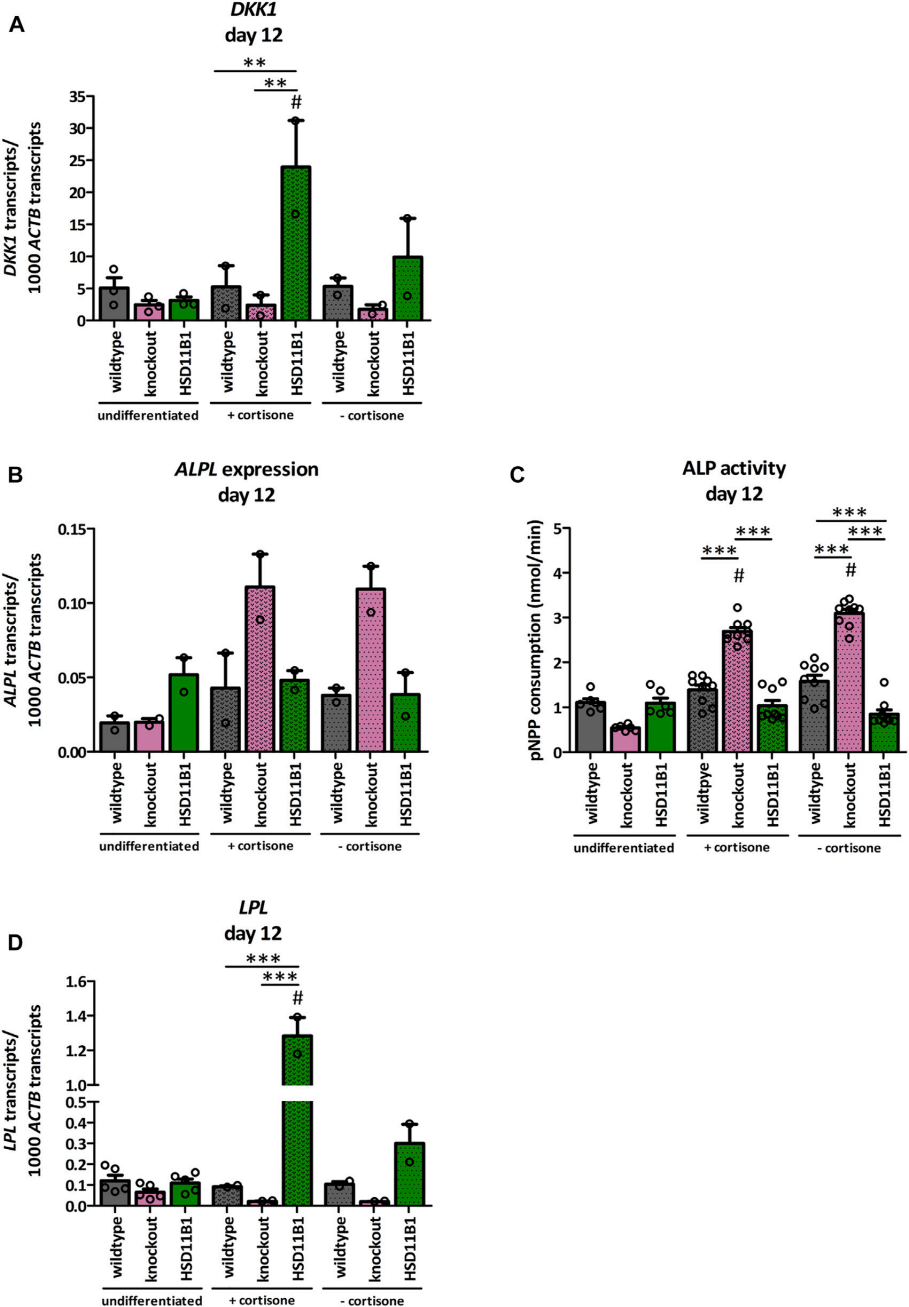
Analyses of the effects of *HSD11B1* knockout and overexpression on osteogenic differentiation. mRNA expression of **(A)** the Wnt signaling marker *DKK1*, **(B)** the osteogenic marker *ALPL*, and **(D)** the adipogenic marker *LPL*. Shown are means ± SEM of 2 independent differentiation experiments (*DKK1*, *ALPL*, *LPL*) and 2 to 5 independent biological replicates of the undifferentiated cells (*DKK1* 3 replicates, *ALPL* 2 replicates, *LPL* 5 replicates). **(C)** ALP activity. Shown are means ± SEM of 6 wells of undifferentiated and 9 wells of differentiated cells from two independent differentiation experiments. Cells were differentiated with ascorbate, *β*-glycerol phosphate, 1α,25-dihydroxyvitamin D3 with or without cortisone. Mean. *p ≤ 0.05, **p ≤ 0.01, ***p ≤ 0.001; $ *p* ≤ 0.05, §*p* ≤ 0.01, #*p* ≤ 0.001 compared to the respective undifferentiated cells, determined by one-way ANOVA with post-hoc Tukey’s Test.

A dexamethasone-free protocol that included 1α,25-dihydroxyvitamin D_3_ was used to analyze the effects of *HSD11B1* knockout and overexpression on osteogenic differentiation. In presence of cortisone, the expression of the Wnt signaling marker Dickkopf-related protein 1 (*DKK1*) significantly increased in the overexpressing cells compared to both the undifferentiated (7.7-fold, *p* < 0.001) and the differentiated wildtype cells (4.6-fold, *p* < 0.01, Figure 6A). This increase was less pronounced when cortisone was not present in the medium, suggesting cortisone dependent effects of *HSD11B1* in osteogenic differentiation.

More importantly, the expression of the osteogenic marker alkaline phosphatase (*ALPL*) was increased only 2.2-fold in the wildtype but 5.6-fold in the knockout cells when compared to undifferentiated control cells (Figure 6B). This represents a 2.6-fold higher increase in the *HSD11B1* knockout cells. The effects were observed both in the presence and in the absence of cortisone. In accordance with this, also ALP activity was increased only in the *HSD11B1* knockout cells when compared to undifferentiated cells (4.8-fold in the presence of cortisone, *p* < 0.001, Figure 6C). The activity was also independent of the presence of excess cortisone in the medium.

Based on the hypotheses that cortisol production by 11β-HSD1 is sufficient to induce adipogenesis in bone cells, also mRNA expression of the adipogenic marker *LPL* was analyzed under osteogenic differentiation (Figure 6D). In *HSD11B1* overexpressing cells and in presence of cortisone, *LPL* expression increased 11.7-fold compared to the undifferentiated controls (*p* < 0.001). This effect was also cortisone dependent (4.3-fold higher *LPL* expression when cortisone was present in the differentiation medium). In contrast, there was no detectable increase in the wildtype cells. In the *HSD11B1* knockout cells, even a decrease in the expression of *LPL* was observed under these conditions. Taken together, these results suggest that *HSD11B1* overexpression may promote adipogenic whereas *HSD11B1* knockout may promote osteogenic differentiation.

## Discussion

Local cortisol metabolism plays a critical role in bone tissue homeostasis (Hardy et al., 2008; Lavery et al., 2012; Morgan et al., 2014; Hardy et al., 2018; Fenton et al., 2021). Here, we generated cell models with stable knockout and overexpression of *HSD11B1* to analyze the impact of local cortisol production by 11β-HSD1 on differentiation of hMSCs.

With the generated cell lines, it is possible to overcome the limitations of transient overexpression and pharmacological inhibition. Properly validated and characterized immortalized cells deliver consistent results and are suitable for initial analyses of molecular mechanisms compared to primary MSCs whose isolation, growth and characterization is time and cost intensive. Moreover, the functionality, i.e., stem cell characteristic of primary MSCs, highly depends on *in vitro* age (Bonab et al., 2006) which makes it particularly difficult to obtain reliable functional outcomes following long-term cultures such as those required for the genetic modifications shown here. We demonstrated the ability of the cells to differentiate into the adipogenic and osteogenic lineage proving the preservation of multipotency of the SCP-1 cells. Furthermore, we generated a SCP-1 cell line carrying a target sequence for flippase-based targeted chromosomal integration and successfully applied these cells. When addressing further scientific questions, these cells could be easily used for stable transfection and overexpression of other genes to generate gain-of-function models. Recently, we modified the Flp-In system to enable targeted integration of multiple genes (Jensen et al., 2020).

In none of the sequenced CRISPR-Cas9 off-target sites, sequence modifications were detected, indicating a high specificity of the used crRNA and thus a specific knockout of *HSD11B1*. Knockout in exon 5 was more efficient than in exon 2. This may result from a lower estimated efficiency of the crRNA targeting exon 2 when compared to the efficiency of the crRNA targeting exon 5. To our knowledge, nothing is known about differences in general target efficiency between exons of the same gene. We could not detect nonsense mediated mRNA decay (NMD) as the premature stop codon in the clone 1C4 is located downstream of the 3′-most exon-exon junction (Nagy und Maquat 1998; Popp und Maquat 2016).

We aimed to generate a knockout cell line from a clonally derived cell clone as mixed clones increase the variability and thereby the probability of inconsistent results. Eight exon 5 clones exhibited more than two types of modifications (Figure 2B) that could be explained by one of the three following points: 1) polyploidy of the analyzed cell clones, 2) a methodological artefact, or 3) the presence of a mixture of cells rather than a single cell clone. Upon generation, SCP-1 cells were shown to be diploid (Böcker et al., 2008) which makes us exclude this possibility. To our knowledge, no other studies on chromosome number have been performed since then. Alternatively, additional sequence modifications may have been detected due to sequencing errors. However, as only sequence clusters with more than 50 reads were analyzed, we would exclude this possibility, too. In our opinion, the most probable scenario is the propagation of cell mixtures rather than single cell clones. In our hands, SCP-1 cells did not grow clonally very well, so that the dilution factor was chosen to be higher than the recommended 0.5 cells/well. After seeding, the cells were strictly observed to identify clones originating from more than one cell. However, despite careful observation of the cells, undocumented mixed clones may have arisen. This proves the necessity of proper genetic validation and the indispensability of deep sequencing.

We applied the here generated *HSD11B1* knockout and overexpression SCP-1 cells to analyze the role of local cortisol in adipogenic and osteogenic differentiation. The lack of a glucocorticoid stimulus in the knockout cells did not abolish lipid droplet formation. This makes the remaining substances of the adipogenic differentiation cocktail (insulin, IBMX and indomethacin) sufficient to stimulate adipogenic differentiation which was also shown in mouse preadipocytes (Park und Ge 2017). *HSD11B1* overexpressing cells formed significantly more lipid droplets and expression of the adipogenic marker *LPL* increased when compared to wildtype or knockout cells, indicating a promoting role of 11β-HSD1 in adipogenic differentiation. Indeed, production of cortisol by 11β-HSD1 was shown to promote adipogenesis in committed omental preadipocytes (Bujalska et al., 2002) and subcutaneous preadipocytes even in the presence of indomethacin, IBMX and insulin (Bujalska et al., 2008). In contrast to our expectations, also in the presence of dexamethasone, the overexpressing cells showed increased lipid droplet formation and *LPL* expression. As dexamethasone is not a substrate of 11β-HSD1 but of 11β-HSD2 (Best et al., 1997; Hult et al., 1998), analyses on the expression and activity of 11β-HSD2 should be considered in further investigations. 11β-HSD2 converts dexamethasone to 11-dehydrodexamethasone which is a substrate for both isozymes *in vitro* (Best et al., 1997) and can therefore be reactivated to dexamethasone by 11β-HSD1 (Best et al., 1997; Rebuffat et al., 2004). Moreover, dexamethasone might induce the expression of *HSD11B2* in SCP-1 cells as it was shown for lung and placental cells (Suzuki et al., 2003; van Beek et al., 2004).

Following osteogenic differentiation, *ALPL* expression and ALP activity was increased in the knockout cells, but cortisone independently. The origin of these cortisone independent effects needs to be investigated in further analyses. *DKK1* expression significantly increased in *HSD11B1* overexpressing cells in the presence of cortisone. Wnt signaling is important for osteoblastogenesis and as *DKK1* is an inhibitor of Wnt signaling (Fedi et al., 1999; Krupnik et al., 1999; Semënov et al., 2005), its increase indicates a reduced osteogenic differentiation. Additionally, expression of the adipogenic marker *LPL* increased in overexpressing cells when cortisone was added to the differentiation medium. It was previously reported that cortisol induces adipogenic differentiation of stromal cells (Pereira et al., 2002). Our data show that high amounts of cortisol generated by enzyme activity of the overexpressed *HSD11B1* promote the switch from osteogenic to adipogenic differentiation. This is in line with our previous observations (Blaschke et al., 2021).

In this study, a limited number of adipogenic and osteogenic markers were analyzed to obtain first results on the effect of 11β-HSD1 on MSC differentiation. Further analyses are needed to investigate the effects of *HSD11B1* knockout and overexpression on adipogenic and especially on osteogenic differentiation in more detail, e.g., its impact on specific matrix generation in microphysiological bone models (Schoon et al., 2020).

We previously showed that suppressed cortisol levels are associated with bone mineral density in patients evaluated for osteoporosis (Siggelkow et al., 2014) indicating a role for 11β-HSD1 in the development of age-related osteoporosis. *HSD11B1* expression increases with age (Cooper et al., 2002; Tiganescu et al., 2011). Therefore, accelerated local cortisol regeneration by increased 11β-HSD1 levels would result in increased adipogenesis in bone and could contribute to the development of age-related osteoporosis. In osteoblasts from subjects aged between 50 and 59 years, expression of adipogenic markers was detected (Clabaut et al., 2021). Specific inhibition of 11β-HSD1 was shown to decrease adipogenesis *in vitro* (Blaschke et al., 2021), to promote osteogenic differentiation and to improve bone microstructure and density *in vivo* (Park et al., 2014; Li et al., 2020). It was further shown that co-culturing of osteoblasts derived from MSCs with bone marrow adipocytes promoted transdifferentiation and increased the expression of adipogenic markers, including *HSD11B1*, in the osteoblasts (Clabaut et al., 2010; Clabaut et al., 2021). This raises the question of whether endogenous cortisol produced by 11β-HSD1 in the adipocytes is involved in this process. Therefore, future analyses should comprise *ex vivo* analyses of 11β-HSD1 expression in human bone and bone marrow.

In conclusion, local cortisol production by 11β-HSD1 had an only limited influence on adipogenic differentiation of MSCs *in vitro*, whereas it clearly impacted osteogenic differentiation. While knockout of *HSD11B1* promoted the osteogenic differentiation, local cortisol production by 11β-HSD1 promoted a shift from osteogenic to adipogenic differentiation. This indicates a role for 11β-HSD1 in the development of osteoporosis by increasing adipogenesis in bone. Taken together, the here generated *HSD11B1* loss-of-function and gain-of-function cell models provide a novel and powerful tool for analyzing the role of 11β-HSD1 in the onset of age-related osteoporosis and could help to identify new therapeutic strategies.

## Data Availability

The raw data supporting the conclusions of this article will be made available by the authors, without undue reservation.
