# Supplementary material for: Effects of HSD11B1 knockout and overexpression on local cortisol production and differentiation of mesenchymal stem cells
Source: Front Bioeng Biotechnol. 2022 Aug 25;10:953034. doi: 10.3389/fbioe.2022.953034 (PMC9453430; doi:10.3389/fbioe.2022.953034)
Supplement: Supplementary file 1 [file DataSheet1.PDF]

Supplementary Material

1 Supplementary Figures and Tables

1.1 Supplementary Figures

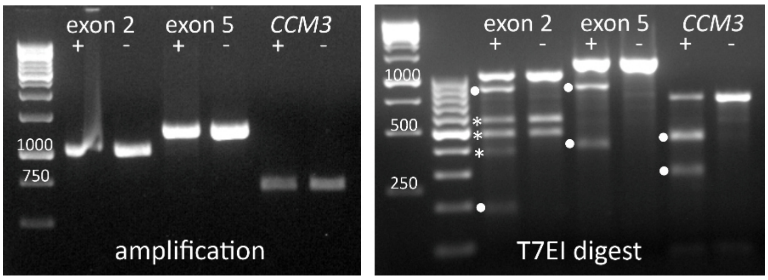

Supplementary Figure 1. T7EI assay to estimate the indel frequency after CRISPR-Cas9 treatment. The regions of interest were amplified by PCR (left). The PCR product was digested with T7EI (right). Expected cleavage products are marked with filled circles. Asterisked bands were not expected. The two larger fragments formed due to the SNP rs5780533 at position 24170 of the *HSD11B1* gene (chr1:209705373, GRCh38.p12). As control, cells were also treated with a crRNA targeting exon 3 of *CCM3* (Schwefel et al., 2018). The crRNA was a kind gift from S. Spiegler and U. Felbor (Department of Human Genetics, University Medicine Greifswald). L, 1 kb DNA ladder; l, 100 bp DNA ladder; fragment sizes of the ladder are given in bp, (+) cells were transfected with a specific crRNA, (-) cells were transfected with the negative control crRNA.

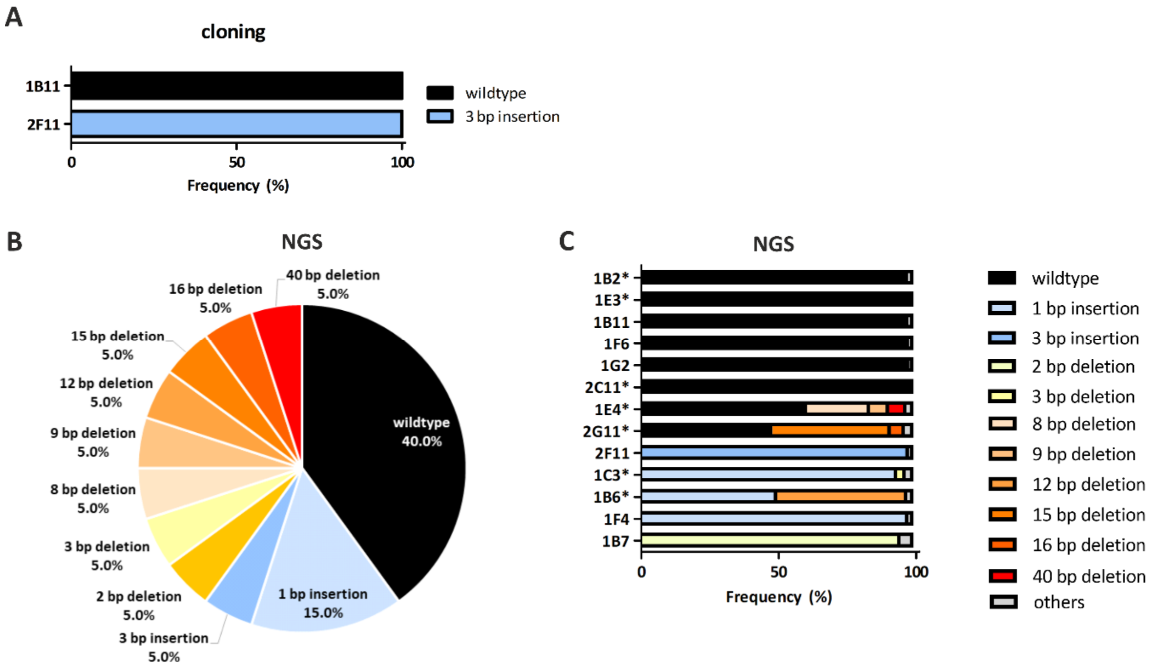

Supplementary Figure 2. Genetic validation of *HSD11B1* knockout SCP-1 cells generated using the crRNA targeting exon 2. (A) Read frequencies of sequence modifications determined by cloning and subsequent capillary sequencing. Due to low cloning efficiency only two clones were analyzed. (B) Summarized allele frequencies of sequence modifications detected by NGS. (C) Read frequencies of sequence modifications across all clones determined by NGS. *Others* represents the clusters that were not analyzed in detail due to a read number < 50.

```
                    10        20        30        40        50        60        70        80        90       100
           ....|....|....|....|....|....|....|....|....|....|....|....|....|....|....|....|....|....|....|....|
Reference  MAFMKKYLLPILGLFMAYYYYSANEEFRPEMLQGKKVIVTGASKGIGREMAYHLAKMGAHVVVTARSKETLQKVVSHCLELGAASAHYIAGTMEDMTFAE
Allele 1   ....................................................................................................
Allele 2   ....................................................................................................

                   110       120       130       140       150       160       170       180       190       200
           ....|....|....|....|....|....|....|....|....|....|....|....|....|....|....|....|....|....|....|....|
Reference  QFVAQAGKLMGGLDMLILNHITNTSLNLFHDDIHHVRKSMEVNFLSYVVLTVAALPMLKQSNGSIVVVSSLAGKVAYPMVAAYSASKFALDGFFSSIRKE
Allele 1   ..............................................LRGPDCSC.AHAEAEQWKHCCRLLSGWESGLSNGCCLFCKQVCFGWVLLLHQ.G
Allele 2   ..............................................GPDCSCL.HAEAE.WKHCCRLL.GWESGLSNGCCLFCKQVC.GWVLLLHQKGIF

                   210       220       230       240       250       260       270       280       290
           ....|....|....|....|....|....|....|....|....|....|....|....|....|....|....|....|....|....|..
Reference  YSVSRVNVSITLCVLGLIDTETAMKAVSGIVHMQAAPKEECALEIIKGGALRQEEVYYDSSLWTTLLIRNPCRKILEFLYSTSYNMDRFINK
Allele 1   IFSVQGQCINHSLCSWPHRHRNSHEGSFWDSPYASSS.GGMCPGDHQR.SSAPRRSVL-------------------------------------
Allele 2   SVQGQCINHSLCSWPHRHRNSHEGSFWDSPYASSSKGGMCPGDHQRGSS.P.RSVL---------------------------------------
```

Supplementary Figure 3. Comparison of 11β-HSD1 peptide sequences of the *HSD11B1* knockout clone 1C4 with the reference sequence. Amino acids are shown in one letter code. Dots represent amino acids that are identical to the reference P28845 (UniProtKB), hyphens represent missing amino acids. The peptide sequence used for targeted proteomics is marked with an orange box.

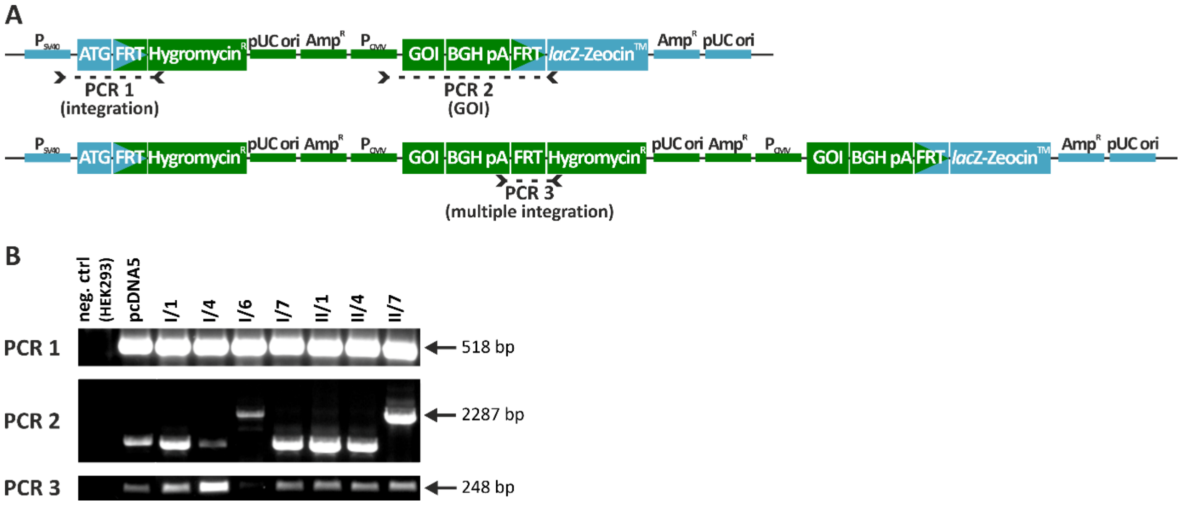

Supplementary Figure 4. Genetic validation of *HSD11B1* overexpressing cells. (A) Schematic representation of binding sites of the PCR primers for validation of the expression cell lines. The targeted integration of the vector into the host cell genome was controlled by PCR 1. The presence of the gene of interest (GOI), *HSD11B1*, was validated by PCR 2. This PCR product was also sequenced by capillary sequencing. Multiple integrations of the expression vector were identified by PCR 3. (B) Representative images of the validation of the targeted chromosomal integration using the validation PCRs. Shown are HEK293 clones and the respective controls. Applied controls were Flp-In T-REx 293 (HEK293) and Flp-In T-REx 293 stably transfected with empty pcDNA5/FRT expression vector (pcDNA5).

1.2 Supplementary Tables

Supplementary Table 1. Sequences of the primers used in this work.

**Primer**	**Direction**	**Sequence 5'-3'**
Amplification of *HSD11B1* mRNA		
HSD11B1	forward	GTCTTCAAGCTTGCTCCCTGTCGGATGGCTTTTA
	reverse	TCCCTCAGATATCCCAGCCCTCAGGGAGTTCCTA
Validation PCR		
PCR 1	forward	AGCTGTGGAATGTGTGTCAGTTAGG
	reverse	ACGCCCTCCTACATCGAAGCTGAAA
PCR 2	forward	CCTTCCTGTAGCCAGCTTTCATCAA
	reverse	CCATGGTGATGCGGTTTTGGCAGTA
PCR 3	forward	AATCGGGGGCTCCCTTTAGGGTTCC
	reverse	ACGCCCTCCTACATCGAAGCTGAAA
T7EI assay		
Ex2_T7EI_PCR	forward	CCGTCCCTGATGTCACAATTCAGAG
	reverse	TGCTGGCCCCTGTGACAATCACTTT
Ex5_T7EI_PCR	forward	TACCCCCCAAAAATCTGCAGCTAAG
	reverse	TAGCCCACTTATCAGACACCTGTGT
Capillary Sequencing		
Ex2_T7EI	forward	CCGTCCCTGATGTCACAATTCAGAG
	reverse	AGCCCAATTAGCTGCTGAGTTGTGA
Ex5_T7EI	forward	TACCCCCCAAAAATCTGCAGCTAAG
	reverse	CAGCCCCTCAAGTCCCTTTGACCTT
Seq_ATRNL1	forward	ACACCCAATAGGCATATGTAAAGGA
	reverse	CTGACGGGCATTTTGAGTAATACCA
Seq_C1orf35	forward	CGGGTGGCCTGCCCTACTTTACTTC
	reverse	GCAGGCCCCATCCAGATGAAGCCAC
Seq_FMNL3	forward	CTTCCCCTTTTCCTCACATCTCCTA
	reverse	CCCCAAGCTCCAGCATTCTCAGAAG
Seq_LTBP1	forward	GCTGACGCTCAAGCTAATTAACACT
	reverse	CGGGCATAAGATAAATTATGAAGAC
Seq_ZRANB1	forward	CTTGGGGTGGTGAGGATGAACATTT
	reverse	GGGTGGTGTCACAAGTAACTTTACA
Next Generation Sequencing		
Ex2_NG	forward	TCGTCGGCAGCGTCAGATGTGTATAAGAGACAGGCTCCCTGTCGGATGGCTTTTATGA
	reverse	GTCTCGTGGGCTCGGAGATGTGTATAAGAGACAGAGCCCAATTAGCTGCTGAGTTGTGA
Ex5_NG	forward	TCGTCGGCAGCGTCAGATGTGTATAAGAGACAGGGTATCAACCCCAGATGATTTCTTA
	reverse	GTCTCGTGGGCTCGGAGATGTGTATAAGAGACAGCAGCCCCTCAAGTCCCTTTGACCTT

Introduced restriction sites are underlined.

Supplementary Table 2. HPLC solvent gradient used for targeted proteomics

Step	Total time (min)	Flow rate (µl/min)	A (%)	B (%)
0	2.00	200	2.0	98.0
1	3.00	200	2.0	98.0
2	40.00	200	25.0	75.0
3	40.10	200	25.0	75.0
4	53.00	200	50.0	50.0
5	53.10	200	60.0	40.0
6	56.00	200	60.0	40.0
7	56.10	200	2.0	98.0
8	65.00	200	2.0	98.0

Supplementary Table 3. MS detection parameters used for targeted proteomics

Protein	Q1	Q3.1	Q3.1-CE	Q3.2	Q3.2-CE	Q3.3	Q3.3-CE	Q3.4	Q3.4-CE	DP	EP	CXP
11β-HSD1	706.9	401.5	23	649.0	33	902.7	26	716.5	31	140	10	13
Na^+/K^+-ATPase	328.3	435.2	15	391.7	14	669.3	15			100	10	13

Q, quadrupole; CE, collision energy; DP, declustering potential; EP, entrance potential; CXP, collision cell exit potential

Supplementary Table 4. Substrate-specific experimental conditions and MS detection parameters for cortisone and cortisol detection Cortisol-D_4 was used as internal standard.

**Substance**	**Mass (Da)**	**Time (min)**	**Organic Solvent**	**DP**	**CE**	**CXP**	**CAD**	**CUR**	**Gas 1**	**Gas 2**	**Temp**	**ISV**
Cortisone	361.1>163.0	3.05	45%	90	34	10	8	25	50	50	450	5500
Cortisol	363.1>121.2	3.12	45%	80	35	10	8	25	50	50	450	5500
Cortisol-d_4	367.0>121.0	3.10	45%	95	37	10	8	25	50	50	450	5500

Time, retention time; Organic Solvent, organic solvent in mobile phase; DP, declustering potential; CE, collision energy; CXP, collision cell exit potential; CAD, collision gas; CUR, curtain gas; Temp, temperature; ISV, ion spray voltage
